# Initial clinical experience with the balloon-in-basket pulsed field ablation system: acute results of the VOLT CE mark feasibility study

**DOI:** 10.1093/europace/euae118

**Published:** 2024-05-03

**Authors:** Prashanthan Sanders, Stewart Healy, Mehrdad Emami, Emily Kotschet, Amber Miller, Jonathan M Kalman

**Affiliations:** Centre for Heart Rhythm Disorders, University of Adelaide and Royal Adelaide Hospital, 1 Port Road, Adelaide, South Australia 5000, Australia; Department of Cardiology, Victorian Heart Hospital, Clayton, Victoria, Australia; Centre for Heart Rhythm Disorders, University of Adelaide and Royal Adelaide Hospital, 1 Port Road, Adelaide, South Australia 5000, Australia; Department of Cardiology, Victorian Heart Hospital, Clayton, Victoria, Australia; Abbott, Minneaplolis, Minnesota, USA; Department of Cardiology, University of Melbourne, Royal Melbourne Hospital and Baker Institute, Parkville, Victoria, Australia

**Keywords:** Atrial fibrillation, Pulsed field ablation, Balloon, Basket, Single shot

## Abstract

**Aims:**

Pulsed field ablation (PFA) for the treatment of atrial fibrillation (AF) potentially offers improved safety and procedural efficiencies compared with thermal ablation. Opportunities remain to improve effective circumferential lesion delivery, safety, and workflow of first-generation PFA systems. In this study, we aim to evaluate the initial clinical experience with a balloon-in-basket, 3D integrated PFA system with a purpose-built form factor for pulmonary vein (PV) isolation.

**Methods and results:**

The VOLT CE Mark Study is a pre-market, prospective, multi-centre, single-arm study to evaluate the safety and effectiveness of the Volt^™^ PFA system for the treatment of paroxysmal (PAF) or persistent AF (PersAF). Feasibility sub-study subjects underwent phrenic nerve evaluation, endoscopy, chest computed tomography, and cerebral magnetic resonance imaging. Study endpoints were the rate of primary serious adverse event within 7 days and acute procedural effectiveness. A total of 32 subjects (age 61.6 ± 9.6 years, 65.6% male, 84.4% PAF) were enrolled and treated in the feasibility sub-study and completed a 30-day follow-up. Acute effectiveness was achieved in 99.2% (127/128) of treated PVs (96.9% of subjects, 31/32) with 23.8 ± 4.2 PFA applications/subject. Procedure, fluoroscopy, LA dwell, and transpired ablation times were 124.6 ± 28.1, 19.8 ± 8.9, 53.0 ± 21.0, and 48.0 ± 19.9 min, respectively. Systematic assessments of initial safety revealed no phrenic nerve injury, pulmonary vein stenosis, or oesophageal lesions causally related to the PFA system and three subjects with silent cerebral lesions (9.4%). There were no primary serious adverse events.

**Conclusion:**

The initial clinical use of the Volt PFA System demonstrates acute safety and effectiveness in the treatment of symptomatic, drug refractory AF.

## Graphical Abstract

**Graphical abstract:**
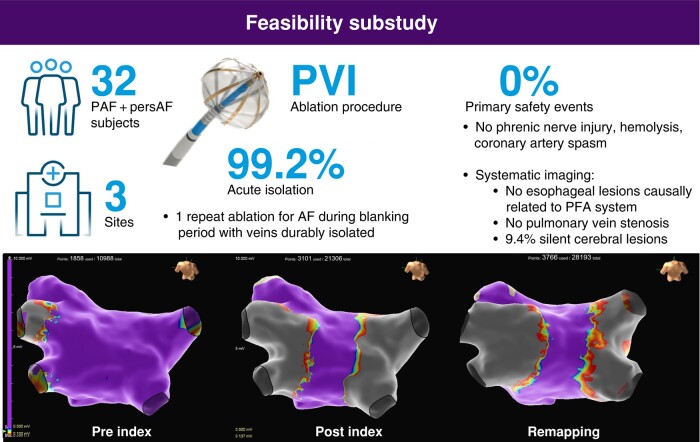

**What’s new?**

- A balloon-in-basket pulsed field ablation system is effective for isolation of the pulmonary veins.
- In this first-in-man experience, there were no significant serious adverse events.
- Potential advantages include greater apposition to the tissue to result in transmural and permanent lesions in fewer applications.

## Introduction

Pulsed field ablation (PFA) is increasingly being utilized for atrial fibrillation (AF) ablation. Although first-generation systems were purported to result in durable pulmonary vein isolation (PVI),^1^ with increasing real-world usage, the success rate of ablation has matched those achieved with thermal-based ablation technologies.^2–8^ The improved safety profile, particularly for collateral injury due to the energy source, has been consistently reported.^2,3^ Importantly, however, new risks, such as coronary artery spasm and haemolysis, are emerging with the wider use of PFA for catheter ablation of AF.^9,10^

Several factors have emerged to determine the achievement of irreversible electroporation and transmurality. These include factors of the energy utilized (field strength, waveform, and number of pulses), catheter design, and tissue proximity.^11,12^ In this context, The Volt^™^ PFA System was developed as a novel, fully 3D-integrated PFA ablation system with a purpose-built form factor for PVI designed for safe and effective treatment of AF. In this study, we present the acute and 30-day safety and efficacy of the first-in-man experience using the Volt system.

## Methods

### Study design

The VOLT CE Mark Study (NCT06106594) is a pre-market, prospective, single-arm, global study to demonstrate safety and effectiveness of the investigational Volt PFA System for the treatment of symptomatic, recurrent, drug-refractory paroxysmal (PAF) and persistent AF (PersAF). The study included a feasibility sub-study in which subjects undergo additional imaging assessments to confirm acute safety and effectiveness after initial clinical use. The sub-study was conducted at three sites in Australia (Royal Adelaide Hospital and Ashford Hospital, Royal Melbourne Hospital, and the Victorian Heart Hospital). The study was approved by the Central Human Research Ethics Committee of the Central Adelaide Local Health Network and corresponding research governance at each institution. The VOLT CE Mark study was sponsored by Abbott.

### Patient selection

The study included subjects undergoing *de novo* AF ablation for symptomatic PAF or PersAF who were refractory, intolerant, or contraindicated to at least one Class I–IV anti-arrhythmic drug. Detailed inclusion and exclusion criteria are available in the Supplementary material online, material. In brief, subjects needed to be over the age of 18 without a prior history of left atrial ablation and without contraindication to an AF ablation. All patients with an implantable cardiac device were excluded.

### Ablation system

The investigational system is comprised of the 12.5-F, over-the-wire, balloon-in-basket PFA catheter (Volt^™^ PFA Catheter Sensor Enabled^™^, Abbott), the 13-F steerable sheath (Agilis^™^ NxT Steerable Introducer Dual-Reach^™^), the custom PGA generator (Volt^™^ PFA Generator), and a compatible electro-anatomical mapping system (EnSite^™^ X EP System EnSite^™^ Pulsed Field Ablation Module). There are three magnetic sensors on the catheter, one distal and two proximal to the balloon, as well as two shaft electrodes proximal to the balloon for visualization on a sensor-enabled mapping system. The catheter balloon is inflated by filling with saline or a saline:contrast mixture for visualization on fluoroscopy. A combination of imaging modalities such as fluoroscopy guidance, electro-anatomic mapping, and intra-cardiac echocardiography can be used to assess catheter position and apposition at the opening of the pulmonary vein for PVI ablation.

The catheter basket consists of eight equally spaced, insulated nitinol splines with a 17.7 mm-exposed portion that serves as the active electrode. The active portion of the spline runs approximately from the equator to the distal portion of the balloon within the basket, creating an elongated electrode with similar overall surface area to that of standard ring electrodes. The bipolar, biphasic pulsed electric field is delivered around the basket to sequential electrode pairs, with 10 R-wave gated pulse trains per therapy application. Baseline therapy includes two applications of the nominal 1800 V waveform. A separate low-voltage waveform is developed for use in locations where phrenic nerve capture cannot be avoided. The baseline for low-voltage therapy is three applications, in which lesion depth has been demonstrated to be maintained relative to nominal baseline applications in pre-clinical testing.

**Figure 1:**
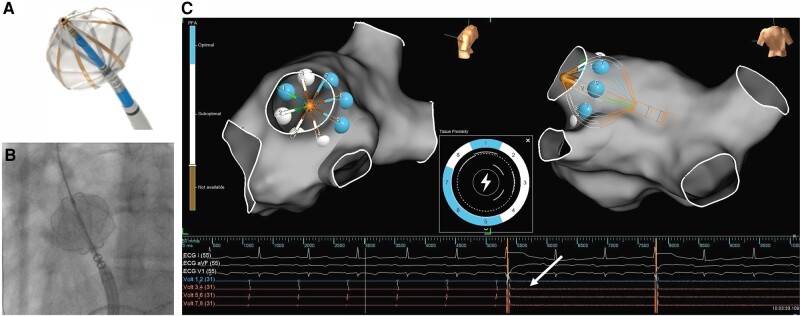
A procedural workflow for pulmonary vein isolation ablation with the Volt™ pulsed field ablation system. (*A*) A balloon-in-basket Volt^™^ pulsed field ablation catheter, Sensor Enabled™. (*B*). A fluoroscopic view of over-the-wire deployment of the balloon-in-basket system in the RSPV, assisted with the use of the sheath. (*C*) Integration of the Volt pulsed field ablation catheter with the Ensite^™^ X EP system. The integration demonstrates the impedance-based tissue proximity indicator displaying real-time electrode-tissue proximity and depicting proximity values via automarks after therapy delivery. An en face visualization in the left image allows accurate rotation to achieve contiguous lesions. The delivered application followed by an attenuation of pulmonary vein signals (arrow) is also seen in the accompanying electrograms.

The electrodes also have pacing, recording, and measuring capabilities, allowing for the detection of local electrical impedance measurements to determine tissue proximity. Dynamic tissue proximity is displayed by the generator and mapping system. *Figure 1* presents the catheter, 3D mapping interface, and proximity indicator.

### Pulsed field ablation procedure

After providing consent and confirming eligibility, subjects underwent an ablation procedure for treatment, for which the Volt PFA System was used. In all cases, the procedure was undertaken without any interruption of oral anticoagulants. The use of general anaesthesia or conscious sedation was allowed per site standard of care. In this cohort, all procedures were undertaken under general anaesthesia. Transoesophageal echocardiography (TOE) was undertaken at the start of the procedure to exclude the presence of thrombus. Ultrasound guidance was required for vascular access. Heparin was administered before trans-septal puncture with a targeted activation clotting time ≥ 350 s throughout the procedure.

**Figure 2:**
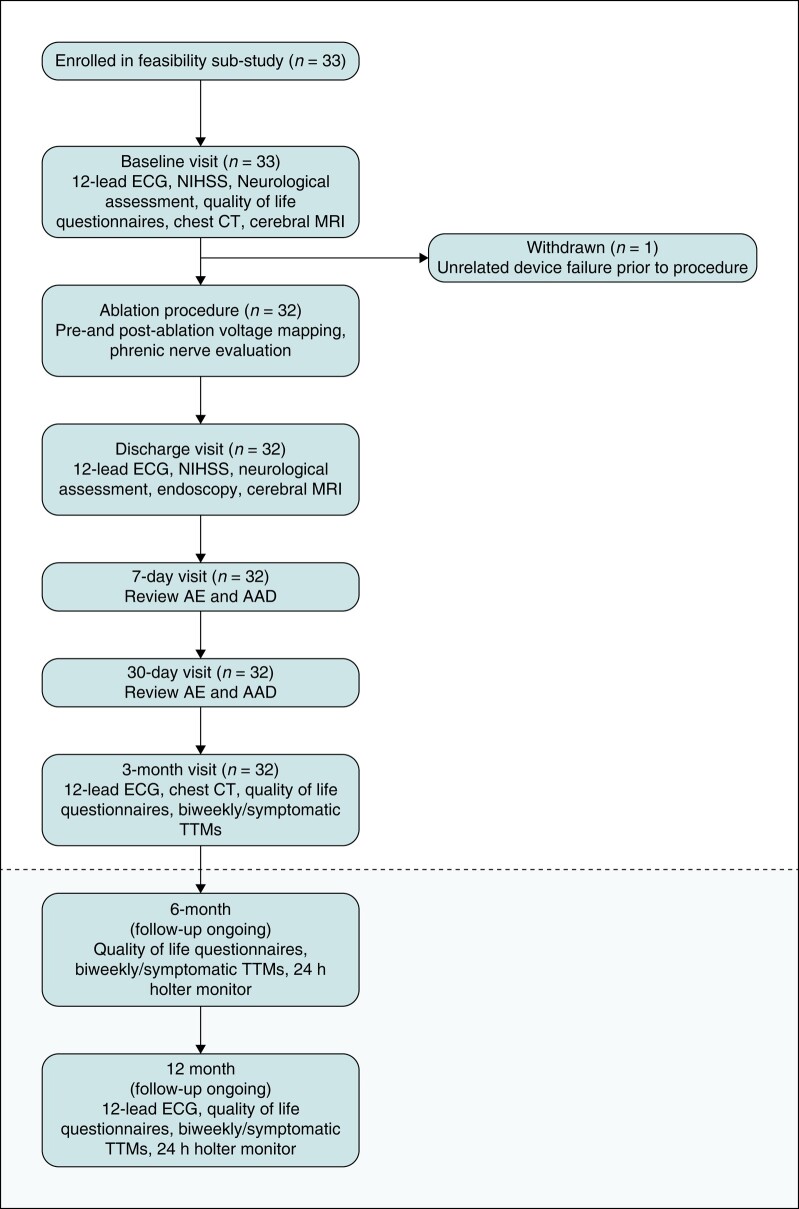
Subject disposition and scheduled events. The presented results pertain to the feasibility sub-study and presents data to 30 days. Below the dotted line depicts the ongoing follow-up and remainder study visits as scheduled for the entire study cohort.

The location of the phrenic nerve was assessed via pacing in the superior vena cava (SVC) prior to trans-septal access; this was annotated on the EnSite system for repeat testing post-ablation. Trans-septal puncture was undertaken with a long sheath and a transeptal needle. Following trans-septal access, a pre-ablation voltage map was generated using a multipolar mapping catheter (HD-Grid). The long transeptal sheath was then exchanged over the wire for the 13-F sheath. The sheath was continuously flushed with heparinized saline. The Volt catheter used an over-the-wire assembly and was introduced through the sheath. Additional pacing to assess phrenic nerve capture was performed with the ablation catheter to mark where capture occurred. The EnSite system and fluoroscopy were utilized to position the catheter sequentially within the PVs and to ensure that ablation was undertaken within the antral region. With optimal positioning of the catheter coaxial to the pulmonary vein, baseline therapy consists of two nominal voltage applications (each application consists of 10 R-wave-gated pulse trains) with a repositioning of the catheter between applications to achieve electrode offset (*Figure 2*). In the right PVs, at each new position, circumferential pacing was undertaken to assess for phrenic nerve capture. If phrenic nerve capture via pacing could not be avoided by repositioning the catheter, the study mandated the application of low-voltage baseline therapy that consisted of three low-voltage applications. Additional therapy applications up to a total of eight applications inclusive of the baseline therapy were allowed per pulmonary vein at the discretion of the physician in order to achieve circumferential pulmonary vein isolation. For these additional applications, nominal voltage could be delivered using electrode selectivity to deselect splines causing phrenic nerve capture so as to not deliver energy at those electrodes.

Ablation was performed targeting electrical isolation of each vein independently. Electrical isolation was confirmed for each PV via entrance block following a minimum 20-min wait period. In the event of residual PV connection, additional therapy applications were delivered up to the maximum allowed treatments per vein if not already applied.

No additional ablations beyond the PVs were allowed using the investigational system, and no additional ablations with other devices were allowed in the left atrium without constituting an effectiveness failure. Ablation to treat cavo-tricuspid isthmus-dependent right atrial flutter that presented during the procedure using a market-approved device was permitted.

Following ablation, a post-ablation voltage map was generated, phrenic nerve function via pacing in the SVC was confirmed, and the absence of pericardial effusion was assessed using trans-thoracic or intra-cardiac echocardiography.

### Follow-up and safety documentation

Prior to the ablation procedure, a review of medical and medication history, a physical examination, and a 12-lead ECG were performed, a neurological assessment was performed by a qualified provider, a National Institutes of Health Stroke Scale (NIHSS) assessment was administered by a certified assessor, and quality-of-life questionnaires were administered as part of the baseline data collection.

At discharge, all subjects underwent a physical examination, 12-lead ECG, and review of medication. The study also required a neurological and NIHSS assessment ≥ 6 h post-procedure prior to discharge. A subject with new findings on the neurologic or NIHSS assessments would be required to have a formal neurological consult and follow-up cerebral magnetic resonance imaging (MRI).

To allow a proactive assessment of oesophageal injury, PV stenosis, and silent cerebral lesions (SCLs), the feasibility sub-study subjects also underwent additional required imaging assessments. These included chest computed tomography (CT) and brain MRI within 30 days prior to the procedure, brain MRI repeated 24–48 h post-procedure, chest CT repeated 3 months’ post-procedure, and an endoscopy 1–3 days post-procedure. If any SCLs were observed, a brain MRI was repeated four or more weeks following the procedure to determine resolution. If any oesophageal lesion was observed, further endoscopy was repeated 2–5 days later to determine progression (indicating non-traumatic lesions). Details of imaging are included in Supplementary material online, material.

Study visits were scheduled to occur 7 days, 30 days, 3 months, 6 months, and 12 months after the index procedure and included a continued review of medications and adverse events (*Figure 2*). A blanking period of 90 days was employed after the index ablation procedure, followed by a 9-month evaluation period for a total of 12 months of follow-up. Left atrial voltage remapping is required for any clinically indicated left atrial repeat ablation during the follow-up period. Medication adjustments, cardioversions, and one repeat ablation procedure utilizing the Volt PFA System could be performed during the blanking period without being considered a long-term effectiveness endpoint failure. Withdrawal of all Class I and III anti-arrhythmic drugs (AADs) was undertaken within 6 weeks after ablation, unless clinically contraindicated. The initial clinical experience reported here presents the data from the feasibility sub-study through the first 30 days of follow-up.

### Primary endpoints

The primary safety endpoint was defined as the rate of subjects experiencing a device- and/or procedure-related serious adverse event with onset within 7 days of any ablation procedure that uses the Volt PFA System from those listed in *Table 3*. All cardiovascular- and procedure-related adverse events were independently adjudicated by a Clinical Events Committee for severity and relatedness.

The acute effectiveness endpoint was defined as the rate of pulmonary veins that were successfully isolated using the Volt PFA system at the end of the index ablation procedure. Acute procedural failure for each pulmonary vein was defined as the inability to isolate the pulmonary vein after maximum allowed therapy applications, assessed after a minimum 20-min waiting period via a confirmation of entrance block at a minimum.

### Statistics

The VOLT CE Mark study was designed to summarize safety and effectiveness using descriptive statistics without formal hypothesis testing or sample size calculations. All endpoints and additional evaluations will be summarized descriptively, with continuous variables summarized as mean ± SD and categorical variables summarized as percentage and counts unless otherwise noted.

## Results

### Study population

A total of 32 subjects (mean age 61.6 ± 9.6 years, 65.6% male, 84.4% PAF) underwent ablation with the investigational system as a part of the VOLT CE Mark feasibility sub-study. Patient enrolment and follow-up is presented in *Figure 2*. One subject was enrolled but withdrawn prior to the insertion of the investigational PFA system due to mechanical bed malfunction. Baseline demographics are summarized in *Table 1*. Feasibility sub-study enrolments through to the 3-month follow-up visits are complete, and longer-term follow-up is ongoing.

**Table 1:** Baseline demographics of the cohort

Patient characteristic	Sub-study Subjects (*n* = 32) % (n/*N*) or Mean ± SD (*n*)
Paroxysmal AF	84.4% (27/32)
Age (years)	61.6 ± 9.6
Gender (male)	65.6% (21/32)
Left ventricular ejection fraction (%)	60.9 ± 7.2
Left atrial diameter (mm)	41.8 ± 5.3
CHA2DS2Vasc score	1.5 ± 1.3
Height (cm)	176.9 ± 7.5
Weight (kg)	96.4 ± 17.4
BMI (kg/m^2^)	30.9 ± 5.6
Cardiovascular history	
New York Heart Association classification	
I	3.1% (1/32)
II	3.1% (1/32)
No heart failure	93.8% (30/32)
Coronary artery disease	12.5% (4/32)
Diabetes	9.4% (3/32)
Hypertension	40.6% (13/32)
Obstructive sleep apnoea	28.1% (9/32)
Stroke	3.1% (1/32)
Transient ischaemic attack	3.1% (1/32)
Thyroid disease	6.3% (2/32)
Anti-arrhythmic medications	
History of Class I/III AAD	84.4% (27/32)
One or more Class I/III AAD ongoing at baseline	78.1% (25/32)
History of Class II/IV/V AAD	78.1% (25/32)

AAD, anti-arrhythmic drug; AF, atrial fibrillation.

### Procedural characteristics

The procedure included required phrenic nerve assessment, pre- and post-procedure voltage mapping, and a 20-min wait period. The procedure and fluoroscopy times in this feasibility sub-study were 124.6 ± 28.1 min and 19.8 ± 8.9 min, respectively. The transpired ablation time (from the first PFA application to the last one) was 48.0 ± 19.9 min, with an ablation catheter left atrial dwell time of 53.0 ± 21.0 min. Procedural characteristics are summarized in *Table 2*.

**Table 2:** Procedure characteristics

Procedure characteristic	Sub-study Subjects (*n* = 32) Mean ± SD (*n*)
Procedure time	124.6 ± 28.1
Fluoroscopy time	19.83 ± 8.89
Left atrial dwell time	53.0 ± 21.0 (32)
Transpired ablation time	48.0 ± 19.9 (32)
Applications per subject	23.8 ± 4.2 (32)
Applications per vein	
LSPV	5.9 ± 1.2 (31)
LIPV	5.6 ± 1.3 (31)
LCPV	8.0 ± NA (1)
RSPV	6.4 ± 1.4 (32)
RIPV	5.8 ± 1.3 (32)
Roof vein	5.0 ± NA (1)

**Table 3:** Safety endpoint events

Endpoint criteria	Sub-study Subjects (*n* = 32)
Atrio-oesophageal fistula	0.0% (0/32)
Cardiac tamponade/perforation	0.0% (0/32)
Death	0.0% (0/32)
Heart block (AV block)	0.0% (0/32)
Myocardial infarction	0.0% (0/32)
Pericarditis	0.0% (0/32)
Phrenic nerve injury resulting in diaphragmatic paralysis	0.0% (0/32)
Pulmonary oedema	0.0% (0/32)
Pulmonary vein stenosis	0.0% (0/32)
Stroke/cerebrovascular accident	0.0% (0/32)
Thromboembolism	0.0% (0/32)
Transient ischaemic attack	0.0% (0/32)
Vagal nerve injury/gastroparesis	0.0% (0/32)
Major vascular access complication/major bleeding	0.0% (0/32)
Device- and/or procedure-related cardiovascular and/or pulmonary adverse event that prolongs hospitalization for more than 48 h	0.0% (0/32)
Total	**0.0% (0/32)**

AV, atrioventricular.

An average of 23.8 ± 4.2 PFA therapy applications were delivered per subject, with 5.9 ± 1.2, 5.6 ± 1.3, 6.4 ± 1.4, and 5.8 ± 1.3 applications per left superior PV, left inferior PV, right superior PV, and right inferior PV, respectively. Phrenic nerve capture via pacing was observed in 10 right-sided veins prior to therapy application, in which the study-mandated low-voltage baseline therapy was delivered with additional therapy applications including a combination of low- and nominal-voltage applications. No transient or persistent phrenic nerve injury was observed in any patient as confirmed by a systematic pacing of the phrenic nerve in the SVC at the completion of the procedure.

### Effectiveness

**Figure 3:**
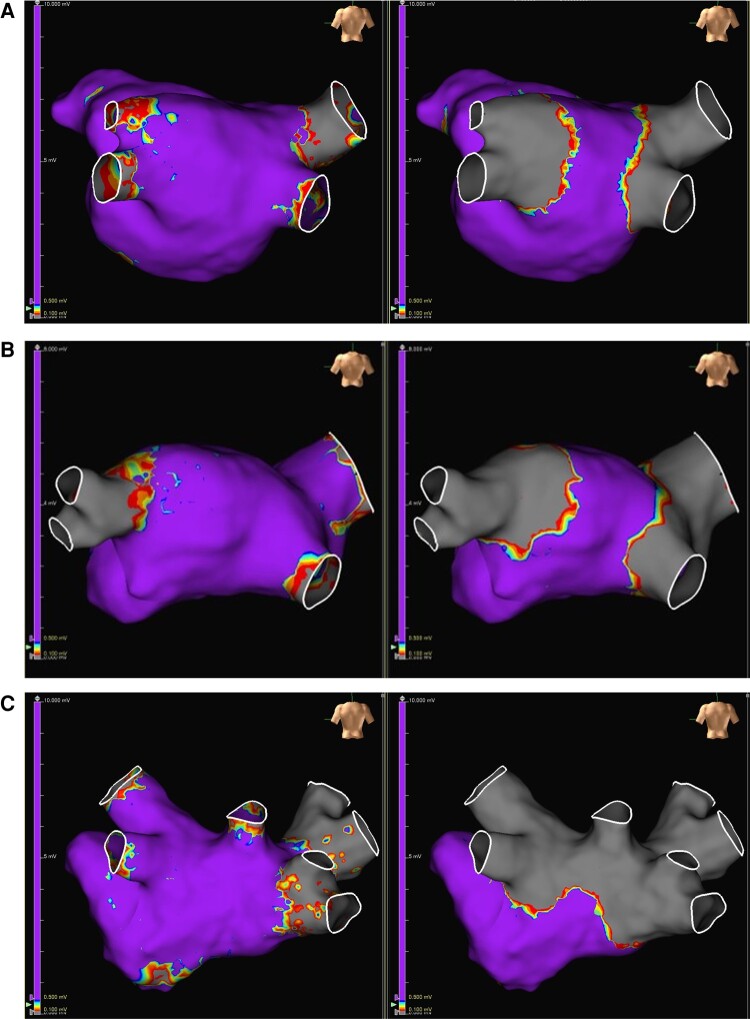
Pre-ablation (left) and post-ablation (right) voltage maps demonstrating the area of pulmonary vein isolation (in grey). Shown are a variety of pulmonary vein morphologies that have been successfully targeted in the feasibility sub-study, including (*A*) typical 4 vein anatomy, (*B*) Left common PV, and (*C*) a roof vein.

Acute effectiveness was achieved in 99.2% (127/128) of treated pulmonary veins (96.9% of subjects, 31/32), including left common PV and roof PV anatomies. An example of pre- and post-procedure voltage maps demonstrating PVI following ablation is shown in *Figure 3*.

An acute reconnection was observed in 1 RIPV of 1 subject after the 20-min waiting period that was successfully isolated after additional therapy applications. There was 1 vein in 1 subject that was unable to be isolated after reaching the protocol-mandated maximum allowed eight applications. In this case, six applications were delivered in the LSPV before starting the 20-min wait period, after which a reconnection was identified using entrance and exit block and appendage mapping. An additional two applications were delivered without successfully isolating the PV.

**Figure 4:**
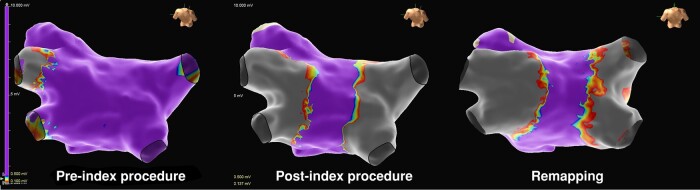
Remapping confirmed durable isolation after more than 60 days post-procedure. Voltage maps from pre-procedure, post-index procedure, and prior to repeat procedure for the single case of left atrial remapping that has been undertaken. This patient had a recurrence of AF. Remapping of the left atrium demonstrated persistent isolation of the pulmonary veins with no regression of the pulsed field ablation area (shown in grey). This patient was treated in the repeat procedure with an isolation of the SVC.

All subjects have completed their 3-month follow-up visit, with long-term follow-up ongoing. At this time, one subject has returned for repeat ablation during the blanking period due to symptomatic recurrence of AF 63 days following the index ablation procedure. Electro-anatomical remapping of the left atrium was performed, which confirmed the persistent isolation of the PVs with demonstratable entrance and exit block. The voltage maps also indicated that the index ablation lesions were maintained without a noticeable regression of the ablation line (*Figure 4*). This subject subsequently underwent ablation in the right atrium only to isolate the superior vena cava. One other subject underwent CTI ablation for right atrial flutter without left atrial mapping during the blanking period.

### Safety

There were no primary safety endpoint events in any subject (*Table 3*). Of note, there were no deaths, clinical strokes, phrenic nerve injury, clinical coronary artery spasm, renal failure, or other SAEs in any subject.

The feasibility sub-study included prospective assessments of oesophageal injury, pulmonary vein stenosis, and silent cerebral lesions (SCLs). There were no oesophageal lesions causally related to the Volt PFA system. Post-procedure endoscopy identified acute oesophageal lesions in three subjects (9.4%) consistent with TOE probe use. Repeat endoscopy confirmed resolution in all subjects, and no subjects experienced any symptoms of atrio-oesophageal fistula. Pulmonary vein diameters from the pre-procedure and 3-month post-procedure chest CT were assessed by independent physicians to assess pulmonary vein stenosis. No instances of pulmonary vein stenosis were observed. Pre- and post-brain MRI were assessed by independent physicians for the presence of SCLs. New SCLs were identified in three subjects (9.4%) following the ablation procedure. The neurological assessments after the procedure showed no manifestations, and repeat MRI confirmed the resolution of all new SCLs. Sub-study results are summarized in *Table 4*.

**Table 4:** Feasibility sub-study imaging results

Feasibility imaging assessments	Sub-study Subjects (*n* = 32)
Brain MRI assessment completed	100.0% (32/32)
New silent cerebral lesion(s) identified	9.4% (3/32)
Number of new silent cerebral lesions identified (*n* = 3)	
Mean ± SD (*n*)	2.3 ± 1.5
Median (Q1, Q3)	2.0, (1.0, 4.0)
Range (min, max)	(1, 4)
Oesophageal assessment completed	100.0% (32/32)
Was any oesophageal injury identified?	9.4% (3/32)
Relatedness to the Volt PFA Catheter	0.0% (0/3)
Symptoms suggestive of atrio-oesophageal fistula identified at follow-up?	0.0% (0/32)
Chest CT assessments completed	93.8% (30^a,b^/32)
Subjects with moderate pulmonary vein stenosis (50–70% narrowing) identified	0.0% (0/30)
LSPV	0.0% (0/29^b^)
LIPV	0.0% (0/30)
LCPV	0.0% (0/1)
RIPV	0.0% (0/31)
RSPV	0.0% (0/31)
Roof vein	0.0% (0/1)
Subjects with severe pulmonary vein stenosis (≥ 70% narrowing) identified	0.0% (0/30)
LSPV	0.0% (0/29^b^)
LIPV	0.0% (0/30)
LCPV	0.0% (0/1)
RIPV	0.0% (0/31)
RSPV	0.0% (0/31)
Roof vein	0.0% (0/1)

CT, computed tomography; MRI: magnetic resonance imaging, PFA, pulsed field ablation; SD, standard deviation.

^a^Post-procedure CT not completed for 1 subject.

^b^Post-procedure CT completed, but LSPV measurements could not be performed due to an oblique angle of image cut-off for 1 subject. This subject was excluded from the subject-level analysis and the PV was excluded from the PV-level analysis.

## Discussion

### Major findings

This study presents the initial experience using the second-generation PFA system, the balloon-in-basket PFA catheter with electroanatomical mapping system integration for AF ablation. It demonstrates the following new information regarding the balloon-in-basket PFA system:

1. The system was highly efficacious for acute PV isolation with rapid and effective isolation of 99.2% of PVs (127/128) regardless of the various PV morphologies.
2. The procedures were performed safely with no primary safety events, including phrenic nerve injury or pulmonary vein stenosis.
3. Systematic endoscopy did not identify any erosions related to PFA.
4. Systematic pre- and post-cerebral MRI identified an acceptable incidence of silent cerebral lesions (not associated with abnormalities on detailed neurological assessment) compared with standard ablation technologies, all of which resolved without sequalae.
5. Finally, in the single case of repeat ablation for AF with remapping that was required to date, there was durable PVI.

### Pulsed field ablation for atrial fibrillation

The initial reports of the use of PFA demonstrated the need for adjustment of unique waveform features to eventually result in what was considered durable PV isolation.^1^ However, with the expansion of use of the optimized waveforms by commercial PFA systems, it has become apparent that in the real world and when used by multiple operators, the success rates of the procedure and the consistency of durable lesions are similar to those observed with thermal energies.^2–8^ While lesion durability may be in part due to specifics of the waveform characteristics, equally there is potential for significant variations in lesion delivery and durability according to catheter features.^1,4,6^ The ‘Basket and Flower’ configuration uses large surface area coverage, while over-the-wire or variable loop catheters provide differing handling capabilities for PVI procedures. Nevertheless, these systems have demonstrated standardization of the procedure across operators and, in some cases, a more expeditious procedure.

The procedural efficiencies anticipated by PFA appear to come to the fore after experience. Early first-in-human experience from the various PFA systems offered modest procedures, LA dwell times, and fluoroscopy times relative to current ablation standards. Such procedural times have been shown to improve with larger clinical studies and real-world experience.^2,3,5,8^ The data presented here summarized the first 32 uses of the Volt PFA System in humans under a highly controlled study design, including mapping the location of the phrenic nerve, phrenic nerve pacing and confirmation, pre- and post-ablation voltage mapping, and a post-ablation 20-min wait period. Procedural characteristics observed in the first subjects using the Volt PFA System are comparable with other early PFA experiences.^4,6^ One notable exception is the current protocol limited therapy delivery to a maximum of eight applications per vein maximum, while early experience with other PFA systems averaged more than eight applications/vein.

The first-generation PFA systems have established the safety of PFA in terms of collateral injury. These have consistently demonstrated the absence of atrio-oesophageal fistula, lung injury, or permanent phrenic nerve injury. However, it is also apparent that the non-energy-specific complications persist with rates of access site complications, embolic events, and cardiac perforation consistent with those seen with established thermal ablation technologies.^2,3^ In addition, with increasing real-world usage, additional unique complications are emerging and include haemolysis with the potential for acute renal failure and coronary artery spasm.^9,10,13^

### The Balloon-in-Basket pulsed field ablation system

The Balloon-in-Basket system has been specifically built for PVI procedures. Increasingly it is recognized that proximity to tissue is an important factor to drive lesion depth with PFA.^11,12,14,15^ The current balloon-in-basket catheter design allows the electrodes to be pushed closer to the tissue surface within the PVs, while insulating the interior of the basket driving energy outward. This, coupled with the impedance-based tissue proximity indicators, potentially facilitates a more transmural delivery of PFA. The balloon-in-basket form factor may also assist in achieving optimal positioning of the electrodes circumferentially around the pulmonary vein to achieve isolation more efficiently than non-balloon-based catheters, as evidenced by the fewer applications required to achieve acute isolation.

In addition, the direct visualization of the catheter on the mapping system provided an appreciation of catheter position in relation to PV and antral anatomy that cannot be obtained with fluoroscopy alone to facilitate contiguous PFA delivery. The acute clinical results and the single case of remapping demonstrating persistent PVI are suggestive that these factors may have contributed to durable PVI.

The negligible lesion regression at remapping relative to the post-procedure voltage map indicates that tissue stunning during the ablation procedure does not appear to over-estimate the low-voltage area achieved from ablation. Similarly, there were two patients in whom acute reconnection was seen within the 20-min waiting period, suggesting that this PFA system may cause less acute tissue stunning such that acute endpoint assessments may be useful.

In the current study, a specific pace mapping protocol was used to identify the location of the phrenic nerve and at these sites delivered a lower dose of PFA. Using this stringent protocol, we did not observe any evidence of phrenic nerve injury, and there was no compromise to the efficacy outcome.

Haemolysis and the potential for renal dysfunction are emerging as a potential new complication of the PFA energy.^10^ The use of the balloon intuitively reduces the flow of blood adjacent to the electrodes and therefore may minimize the haemolytic effect during PFA delivery. Although the presence of haemolysis was not prospectively assessed via systematic baseline and post-procedure assessment in this protocol, no adverse events related to haemolysis or renal dysfunction were reported.

### Feasibility assessments

The study was designed to assess the initial acute safety of the Volt PFA System through systematic imaging to proactively detect the onset of adverse events of interest. The sub-study subjects revealed three non-serious adverse events of oesophageal lesions. At the investigational sites partaking in this feasibility study, TOE was used to guide trans-septal puncture and catheter manipulations. Oesophageal lesions have been identified in 30% of patients undergoing PVI ablation with TOE workflow in a study previously performed by operators on this study.^16^ Similarly, non-serious oesophageal lesions have been observed in over 20% of patients undergoing TOE in the absence of cardiac ablation, and the incidence of oesophageal lesions is known to increase with the duration of TOE use during the performance of the procedure.^16^

The sub-study subjects revealed three non-serious adverse events of silent cerebral lesions. This rate is considered within the anticipated range for a cardiac ablation procedure. Similar PFA feasibility studies have reported rates of silent cerebral lesions between 3.0 and 20.5%,^6,17^, and silent cerebral phenomena occur after AF ablation with multiple ablation technologies, ranging between 2 and 40%.^18–21^

No sub-study subjects had symptoms related to atrio-oesophageal fistula through the 30-day follow-up, and there were no events of atrio-oesophageal fistula, stroke, or transient ischaemic attack. There have also been no reports of pulmonary vein stenosis or phrenic nerve injury through the 30-day follow-up.

### Limitations

The current experience is a first-in-man experience of the initial use of the Volt PFA system. It consisted only of a limited number of proceduralists who were highly supported by the developers of the system. Use of the catheter was limited to pulmonary vein isolation only and no additional therapy targets were allowed, limiting the ability to assess the safety and effectiveness of posterior wall ablation or ablation of other targets with this catheter. Nevertheless, this study demonstrates the safety and acute efficacy of the system for PVI and provides the foundation for more widespread evaluation.

## Conclusions

The Volt balloon-in-basket PFA system is safe and effective for PVI. It expands the armamentarium available to the clinician of PFA for PVI. There are several potential advantages over the currently available systems that require further evaluation in a multicentre study.

## Supplementary Material

euae118_Supplementary_Data

## Data Availability

The data underlying this article will be shared on reasonable request to the corresponding author in accordance with prevailing Human Research Ethics Committee approvals.
