# Supplementary material for: Initial clinical experience with the balloon-in-basket pulsed field ablation system: acute results of the VOLT CE mark feasibility study
Source: Europace. 2024 May 3;26(5):euae118. doi: 10.1093/europace/euae118 (PMC11098042; doi:10.1093/europace/euae118)
Supplement: euae118_Supplementary_Data [file euae118_supplementary_data.docx]

**SUPPLEMENTAL MATERIAL**

**Initial Clinical Experience with the Balloon-in-Basket Pulsed Field Ablation System:**

**Acute Results of the VOLT CE Mark Feasibility Study**

Prashanthan Sanders, MBBS, PhD;^1^ Stewart Healy, MBBS;^2^ Mehrdad Emami, MD, PhD;^1^ Emily Kotschet, MBBS;^2^ Amber Miller, PhD;^3^ Jonathan M. Kalman, MBBS, PhD^4^

**From:** ^1^Centre for Heart Rhythm Disorders, University of Adelaide and Royal Adelaide Hospital, Adelaide, Australia; ^2^Victorian Heart Hospital, Victoria, Australia; ^3^Abbott, Minnesota, USA; ^4^University of Melbourne, Royal Melbourne Hospital and Baker Institute, Victoria, Australia

**Inclusion/Exclusion Criteria**

### Inclusion Criteria

A patient will be eligible for clinical trial participation if he/she meets the following criteria:

1. Documented symptomatic PAF or PersAF. Documentation requirements are as follows:

Paroxysmal:

- Physician’s note indicating recurrent self-terminating AF with ≥ 2 episodes of PAF within the 6 months prior to enrollment **AND**
- One electrocardiographically documented PAF episode within 12 months prior to enrollment.

Persistent: Continuous AF sustained beyond 7 days and less than 1 year that is documented by

- Physician's note, **AND** either
- 24-hour Holter within 180 days prior to enrollment, showing continuous AF, **OR**
- Two electrocardiograms (from any form of rhythm monitoring) showing continuous AF:
  - That are taken at least 7 days apart but less than 12 months apart
  - If electrograms are more than 12 months apart, there must be one or more Sinus Rhythm recordings in between or within 12 months prior to consent/enrollment
  - The most recent electrocardiogram must be within 180 days of enrollment.

NOTE: Documented evidence of the AF episode must either be continuous AF on a 12-lead ECG or include at least 30 seconds of AF from another ECG device.

1. Plans to undergo a PVI catheter ablation procedure due to symptomatic PAF or PersAF and is refractory, intolerant, or contraindicated to at least one Class I-IV AAD medication
2. At least 18 years of age
3. Able and willing to comply with all trial requirements including pre-procedure, post- procedure, and follow-up testing and requirements
4. Informed of the nature of the trial, agreed to its provisions, and has provided written informed consent as approved by the Institutional Review Board/Ethics Committee (IRB/EC) of the respective clinical trial site.

### Exclusion Criteria

A patient will be excluded from enrollment in the clinical trial if he/she meets any of the following criteria:

1. Previously diagnosed long-standing persistent atrial fibrillation (Continuous AF greater than 1 year in duration)
2. Arrhythmia due to reversible causes including thyroid disorders, acute alcohol intoxication, electrolyte imbalance, severe untreated sleep apnea, and other major surgical procedures in the preceding 90 days
3. Patient known to require ablation beyond PVI at the time of consent.
4. Known presence of cardiac thrombus
5. Left atrial diameter ≥ 5.5 cm (anteroposterior diameter) within 180 days of index procedure.
6. Left ventricular ejection fraction < 35% as assessed with echocardiography within 180 days of index procedure
7. New York Heart Association (NYHA) class III or IV heart failure
8. Body mass index > 40 kg/m^2^
9. Pregnant, nursing, or planning to become pregnant during the clinical investigation follow-up period
10. Patients who have had a ventriculotomy or atriotomy within the preceding 30 days of procedure,
11. Myocardial infarction (MI), acute coronary syndrome, percutaneous coronary intervention (PCI), or valve or coronary bypass grafting surgery within preceding 90 days
12. Unstable angina
13. Stroke or TIA (transient ischemic attack) within the last 90 days
14. Heart disease in which corrective surgery is anticipated within 180 days after procedure
15. History of blood clotting or bleeding abnormalities including thrombocytosis, thrombocytopenia, bleeding diathesis, or suspected anti-coagulant state
16. Contraindication to long term anti-thromboembolic therapy
17. Patient unable to receive heparin or an acceptable alternative to achieve adequate anticoagulation
18. Known sensitivity to contrast media (if needed during the procedure) that cannot be controlled with pre-medication
19. Previous left atrial surgical or left atrial catheter ablation procedure (including LAA closure device)
20. Presence of any condition that precludes appropriate vascular access
21. Severe mitral regurgitation (regurgitant volume ≥ 60 mL/beat, regurgitant fraction ≥ 50%, and/or effective regurgitant orifice area ≥ 0.40cm^2^).
22. Previous tricuspid or mitral valve replacement or repair
23. Patients with prosthetic valves
24. Patients with a myxoma
25. Patients with an interatrial baffle or patch as the transseptal puncture could persist and produce an iatrogenic atrial shunt
26. Stent, constriction, or stenosis in a pulmonary vein
27. Rheumatic heart disease
28. Hypertrophic cardiomyopathy
29. Diagnosed with amyloidosis or atrial amyloidosis
30. Active systemic infection
31. Renal failure requiring dialysis
32. Severe pulmonary disease (e.g., restrictive pulmonary disease, constrictive or chronic obstructive pulmonary disease) or any other disease or malfunction of the lungs or respiratory system that produces severe chronic symptoms
33. Presence of an implantable therapeutic cardiac device including permanent pacemaker, biventricular pacemaker, or any type of implantable cardiac defibrillator (with or without biventricular pacing function) or planned implant of such a device for any time during the follow-up period. Presence of an implantable loop recorder is acceptable as long as it is removed prior to insertion of the investigational device.
34. Presence of an implanted LAA closure device or plans to have an LAA closure device implanted during the follow-up period
35. Patient is currently participating in another clinical trial or has participated in a clinical trial within 30 days prior to screening that may interfere with this clinical trial without pre-approval from this study Sponsor
36. Unlikely to survive the protocol follow up period of 12 months
37. Presence of other medical, anatomic, comorbid, social, or psychological conditions that, in the investigator’s opinion, could limit the subject’s ability to participate in the clinical investigation or to comply with follow-up requirements, or impact the scientific soundness of the clinical investigation results.
38. Individuals without legal authority
39. Individuals unable to read or write

**Feasibility Sub-study imaging recommendations**

Strongly recommended guidelines for brain MRI are described below.

Minimal requirements:

- Use of the same imaging equipment, sequences, and specifications is strongly recommended for pre- and post-procedure imaging of the same patient
- 1.5 Tesla MR scanner
- Head coil
- Minimal imaging sequences include: Diffusion weighted sequence and FLAIR sequence (Turbo-spin-echo)

Recommended sequences and specifications:

A) T2- Turbo-spin-echo sequence

- Dark fluid preparation
- TR/TE/flip angle: 9000ms/ 88ms/150°
- Spatial resolution: 0.7x0.7mm
- Slice thickness 5mm (range 4-5mm); match slice thickness with diffusion weighted sequence
- Gap 1.5mm (range 1.5 - 5.6 mm); match gap with diffusion weighted sequence
- Fat sat

B) 2D-diffusion weighted sequence

- TR/TE: 6300ms/ 89ms
- Spatial resolution: 0.6x0.6mm
- Slice thickness 5mm (range 4-5mm); match slice thickness with T2- sequence
- Gap 1.5mm (range 1.5 – 5.6mm); match gap with T2-sequence
- 3 averages
- B=0 and B=1000
- Apparent Diffusion Coefficient map
- Fat sat
